# Bioinformatics analysis of PANoptosis regulators in the diagnosis and subtyping of steroid-induced osteonecrosis of the femoral head

**DOI:** 10.1097/MD.0000000000037837

**Published:** 2024-05-03

**Authors:** Qiang Ding, Bo Xiong, Jinfu Liu, Xiangbin Rong, Zhao Tian, Limin Chen, Hongcheng Tao, Hao Li, Ping Zeng

**Affiliations:** a The First Clinical Medical College, Guangxi University of Chinese Medicine, Nanning, China; b Yulin Orthopedic Hospital of Integrated Traditional Chinese and Western Medicine, Yulin, China; c Ruikang Hospital Affiliated to Guangxi University of Chinese Medicine, Nanning, China; d Guangxi Traditional Chinese Medical University Affiliated First Hospital, Nanning, China.

**Keywords:** biomarkers, immune infiltration, machine learning models, molecular subtypes, PANoptosis, SONFH

## Abstract

In this study, we aimed to investigate the involvement of PANoptosis, a form of regulated cell death, in the development of steroid-induced osteonecrosis of the femoral head (SONFH). The underlying pathogenesis of PANoptosis in SONFH remains unclear. To address this, we employed bioinformatics approaches to analyze the key genes associated with PANoptosis. Our analysis was based on the GSE123568 dataset, allowing us to investigate both the expression profiles of PANoptosis-related genes (PRGs) and the immune profiles in SONFHallowing us to investigate the expression profiles of PRGs as well as the immune profiles in SONFH. We conducted cluster classification based on PRGs and assessed immune cell infiltration. Additionally, we used the weighted gene co-expression network analysis (WGCNA) algorithm to identify cluster-specific hub genes. Furthermore, we developed an optimal machine learning model to identify the key predictive genes responsible for SONFH progression. We also constructed a nomogram model with high predictive accuracy for assessing risk factors in SONFH patients, and validated the model using external data (area under the curve; AUC = 1.000). Furthermore, we identified potential drug targets for SONFH through the Coremine medical database. Using the optimal machine learning model, we found that 2 PRGs, CASP1 and MLKL, were significantly correlated with the key predictive genes and exhibited higher expression levels in SONFH. Our analysis revealed the existence of 2 distinct PANoptosis molecular subtypes (C1 and C2) within SONFH. Importantly, we observed significant variations in the distribution of immune cells across these subtypes, with C2 displaying higher levels of immune cell infiltration. Gene set variation analysis indicated that C2 was closely associated with multiple immune responses. In conclusion, our study sheds light on the intricate relationship between PANoptosis and SONFH. We successfully developed a risk predictive model for SONFH patients and different SONFH subtypes. These findings enhance our understanding of the pathogenesis of SONFH and offer potential insights into therapeutic strategies.

## 1. Introduction

Steroid-induced osteonecrosis of the femoral head (SONFH) is a condition that disrupts blood supply to the femoral head, trabecular bone necrosis, and bone marrow in the femoral head and is a result of long-term or high-dose use of glucocorticoids.^[1,2]^ Currently, the pathogenesis of SONFH is not fully understood; it may be influenced by multiple interrelated factors.^[3]^ Bone metabolism is a complex series of biological processes that involve the synthesis and absorption of bone matrix. Abnormal metabolism can cause bone loss, trabecular collapse, and ultimately osteonecrosis. The stability of bone metabolism depends on the dynamic balance between osteoblasts involved in bone formation and osteoclasts involved in bone resorption, which is strictly regulated by multiple systems of the body.^[4,5]^ Pyroptosis, apoptosis, and necroptosis are the most genetically defined programmed cell death pathways and are closely related to homeostasis and disease.^[6]^ In contrast, PANoptosis is a novel inflammatory programmed cell death pathway regulated by the PANoptosome complex, with the key features of pyroptosis, apoptosis, and necroptosis. Moreover, PANoptosis cannot be characterized by any one of the 3 death modes alone but rather by the interaction among them.^[7,8]^

Current research shows that PANoptosis is involved in the occurrence and development of many diseases, including aseptic inflammation, infection, and cancer.^[9]^ Studies have shown that excessive use of glucocorticoids can lead to bone loss. Osteoblast apoptosis and necroptosis are closely involved in the process.^[10,11]^ In addition, excessive use of glucocorticoids can render osteoblasts more susceptible to oxidative stress stimulation and upregulate the expression of inflammasomes, thereby inducing pyroptosis in osteoblasts and leading to osteogenic dysfunction.^[12]^ These studies suggest that PANoptosis may be closely involved in systemic bone metabolism disorders and osteoporosis induced by excessive use of glucocorticoids, eventually leading to micro-fractures of the femoral head under stress when the patient is weight-bearing and inducing ONFH.^[13,14]^ However, the molecular mechanism of SONFH and PANoptosis remains unclear, and the mechanism by which glucocorticoids affect bone repair and bone homeostasis through PANoptosis requires further investigation. To date, no bioinformatics study has been found on the relationship between PANoptosis and SONFH. Nevertheless, high-throughput sequencing technology is widely used to understand and obtain information about the molecular mechanisms involved in the pathological processes of disease. Li et al^[15]^ collected peripheral serum samples of SONFH patients and control subjects for omics analysis and uploaded the data to the GEO database. This provided data support for the analysis of molecular characteristics related to SONFH. Therefore, in this study, the key genes related to PANoptosis in SONFH were screened using bioinformatics, immune infiltration analysis, and machine learning models, and the diagnostic value of these genes was explored. We hope our study results will reveal possible molecular mechanisms in the pathological process of SONFH and provide potential biomarkers for further investigation of SONFH.

## 2. Materials and methods

### 2.1. Data source and acquisition of differentially expressed PANoptosis-related genes

Ethical approval was not necessary as the study used publicly available data from the GEO database (http://www.ncbi.nlm.nih.gov/geo). GSE123568 was downloaded from the GEO database. The dataset includes 10 control subjects and 30 patients with SONFH, and includes 20 male cases and 20 female cases. Sample tissue was derived from peripheral blood mononuclear cells. After annotation, the original gene expression profile of GSE123568 was normalized and corrected by the “limma” package. PANoptosis-related genes (PRGs) were obtained from a published article,^[16]^ which include *NLRP3, CASP8, PARP1, TAB3, TNFAIP3, TAB2, RIPK1, ZBP1, IRF1, PSTPIP2, AIM2, CASP7, RIPK3, GSDMD, MLKL, CASP1, FADD, TRADD*, and *CASP6*.

The PRG expression matrix was obtained by combining the normalized gene expression matrix. Subsequently, the “reshape2” and “ggpubr” packages were used to analyze and identify PRGs associated with SONFH. Using *P* < .05 as the criterion, differentially expressed PRGs between SONFH patients and control subjects were screened using the wilcox.test. Heat maps and box plots were then created to visualize the results. Finally, the “circus” package was used to visualize the chromosomal localization of the PRGs.

### 2.2. Correlation analysis of differentially expressed PRGs

We used the “corrplot” and “circlize” packages to analyze the interrelationship between differentially expressed PRGs and to perform visualization processes.

### 2.3. Correlation analysis between differentially expressed PRGs and immune infiltration

We obtained gene expression data for immune cells from the CIBERSORT database (https://cibersortx.stanford.edu/). We used the “preprocessCore” package and the CIBERSORT algorithm to analyze the normalized gene expression matrix for immune cell infiltration. We then used the “reshape2” and “ggpubr” packages to compare the expression levels of differentially expressed PRGs between the immune cell subtypes. We used the Spearman correlation coefficient to assess the association between immune cell subtypes and differentially expressed PRGs, and considered *P* < .05 as statistically significant.

### 2.4. Sample clustering and immune infiltration analysis

The GSE123568 dataset samples were genotyped based on differentially expressed PRGs using the “ConsensusClusterPlus” package. The typing results were then analyzed using the “pheatmap,” “reshape2,” and “ggpubr” packages to construct subtype boxplots and heatmaps. To observe the internal similarity of each subtype sample and the differentiation between different subtypes, we used the “limma” and “ggplot2” packages to analyze the typing results and draw principal component analysis scatter plots. Next, we analyzed the typing results and the immune cell infiltration results files using the “limma,” “reshape2,” and “ggpubr” packages to construct box plots.

### 2.5. Gene set variation analysis (GSVA)

To assess pathway enrichment in different clusters, we applied the “GSVA” package. We obtained the file “C2.cp.kegg.symbols” from the MSigDB website database. To identify pathways that were differentially expressed between subtypes, we used the “limma” package to compare GSVA scores. We considered *P* < .05 to be significant using a two-sided *t* test.

### 2.6. Weighted gene co-expression network analysis (WGCNA)

We employed the “WGCNA” package to identify co-expressed modules. To ensure the accuracy of our results, we selected the top 25% of genes with the most variation for subsequent WGCNA analysis. We selected the optimal soft threshold to construct the adjacency matrix and transformed it into a topological overlap matrix (TOM). We set the minimum module size to 100 and used the TOM dissimilarity (1-TOM) based on a hierarchical clustering tree algorithm to obtain modules. Module significance reflects the relationship between modules and disease status, whereas gene significance is defined as the correlation between a gene and a clinical phenotype.

### 2.7. Constructing a machine learning model to screen and verify key predictive genes

The GSE123568 dataset was divided into a training group and a test group at a ratio of 7:3 to construct and verify the classification model, respectively. The generalized linear (GLM), support vector machine (SVM), eXtreme gradient boosting (XGB), and random forest (RF) models were constructed using the “caret,” “DALEX,” “ggplot2,” “randomForest,” “kernlab,” and “xgboost” packages in the training group. The cluster-specific differentially expressed genes (DEGs) were analyzed based on the 4 models, and the residual boxplots and gene importance analysis were obtained. The top 5 genes with importance scores were outputted. The receiver operating characteristic (ROC) curve was drawn using the “pROC” package. The optimal model and its key predictive genes were selected based on the residual boxplot and the area under the curve (AUC) of the ROC curve. The key predictive genes were used to validate the external dataset GSE74089. Additionally, the machine learning and nomogram models were evaluated by drawing the ROC curve of the external data to assess their predictive efficiency.

### 2.8. Correlation analysis between differentially expressed PRGs and key predicted genes

The “corrplot” and “circlize” packages were used to analyze the interrelationship between differentially expressed PRGs and key predicted genes. In the final step, we visualized them.

### 2.9. Drug target prediction of key predicted genes and highly correlated differentially expressed PRGs

We identified key predicted genes related to SONFH and highly correlated differentially expressed PRGs using a key mapping approach. Next, the Coremine medical information retrieval platform (www.coremine.com/medical/) was to analyze the obtained results. After applying a significance level of *P* < .05, we selected potential drug targets for further investigation.

### 2.10. Sample collection

Peripheral blood was collected from 4 patients with hormone-induced femoral head necrosis and 4 patients with femoral neck fracture who underwent joint replacement surgery in the Yulin Orthopedic Hospital of Integrated Traditional Chinese and Western Medicine. All patients strictly read and signed the informed consent form, which was approved by the Ethics Committee of the Yulin Orthopedic Hospital of Integrated Traditional Chinese and Western Medicine. The study follows the guidelines of the 1975 Declaration of Helsinki.

### 2.11. Reverse-Transcription Quantitative Polymerase Chain Reaction (RT-qPCR)

Total RNA was extracted from human peripheral blood according to the instructions of the RNA extraction kit, and then the first strand cDNA was synthesized, and RT-qPCR was performed using cDNA as the template. The reaction system was 20 µL, and the reaction conditions were predenatured at 95°C for 30 seconds, denatured at 95°C for 10 seconds, annealing at 60°C for 30 seconds, and 40 cycles. Using β-actin as the internal reference, the relative expression levels of MLKL and CASP1 mRNA were calculated by 2−ΔΔCt. We used the primer sequences listed in Table 1 to amplify the genes of interest.

**Table 1:** Primer sequences of *GAPDH, MLKL* and *CASP1*.

Gene name	Forward primer	Reverse primer
GAPDH	GGAAGCTTGTCATCAATGGAAATC	TGATGACCCTTTTGGCTCCC
*MLKL*	TTTCTAACAGCAAGCCAGGACA	CTCCTTGGCTTATGGGTGAAA
*CASP1*	TCGCTTTCTGCTCTTCCACA	GGCATCTGCGCTCTACCATCT

### 2.12. Statistical analysis

Values for all data were presented as mean ± standard deviation (SD). Comparison of 2 groups was conducted with Student’s *t* test. *P* values less than .05 were considered to be significant.

## 3. Results

### 3.1. Differentially expressed PRGs in steroid-induced femoral head necrosis

**Figure 1.**
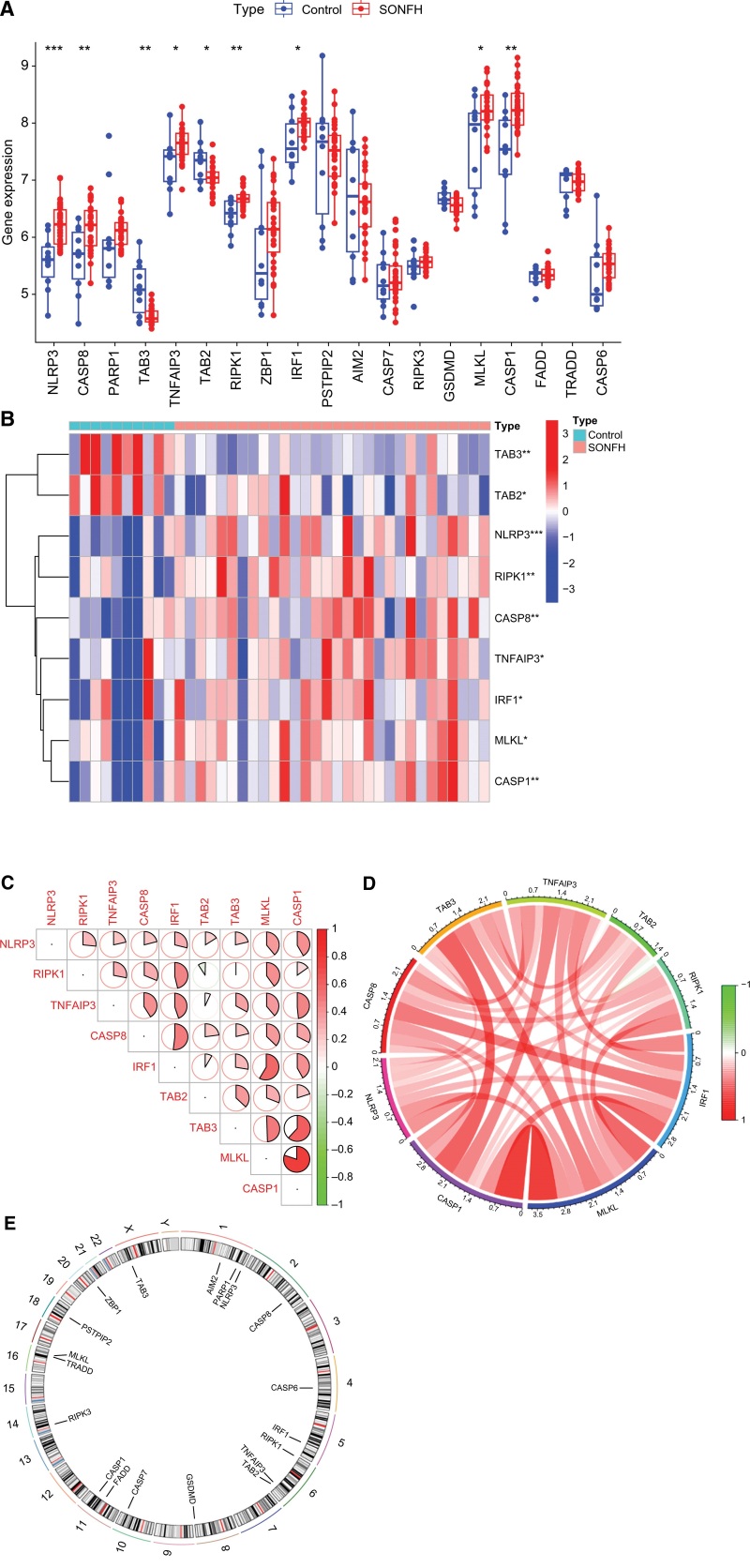
Expression characteristics of differentially expressed PANoptosis-related genes (PRGs) and gene localization of PRGs. (A) Box plot of differentially expressed PRGs between 2 groups. (B) Heatmap of differentially expressed PRGs in 2 groups. (C, D) Spearman correlation analysis of differentially expressed PRGs. (E) Location of PRGs on chromosomes (* *P* < .05, ** *P* < .01, *** *P* < .001).

We extracted a gene expression matrix for 9 differentially expressed PRGs from 30 SONFH patients and 10 control subjects. The results of the boxplots and heat maps (Fig. 1A and B) demonstrated that *NLRP3, CASP8, RIPK1, CASP1, TNFAIP3, IRF1*, and *MLKL* were highly expressed in SONFH patients. However, *TAB3* and *TAB2* were less expressed in SONFH patients (Fig. 1A and B). The sector and circle plots of the analysis of the correlation between differentially expressed PRGs showed that *IRF1* demonstrated a strong synergistic effect with *CASP8* and *MLKL*. There was a strong synergistic effect between *CASP1* and *TAB3*, as well as between *CASP1* and *MLKL*. (Fig. 1C and D). The chromosomal localization of PRGs showed that *AIM2, PARP1*, and *NLRP3* were located on chromosome 1; *CASP8* on chromosome 2; *CASP6* on chromosome 4; *IRF1* on chromosome 5; *RIPK1, TNFAIP3*, and *TAB2* on chromosome 6; and *GSDMD* on chromosome 8. *CASP7* is located on chromosome 10, *CASP1* and *FADD* on chromosome 11, *RIPK3* on chromosome 14, *MLKL* and *TRADD* on chromosome 16, *PSTPIP2* on chromosome 18, *ZBP1* on chromosome 20, and *TAB3* on chromosome X (Fig. 1E).

### 3.2. Immune infiltration characteristics of differentially expressed PRGs

**Figure 2.**
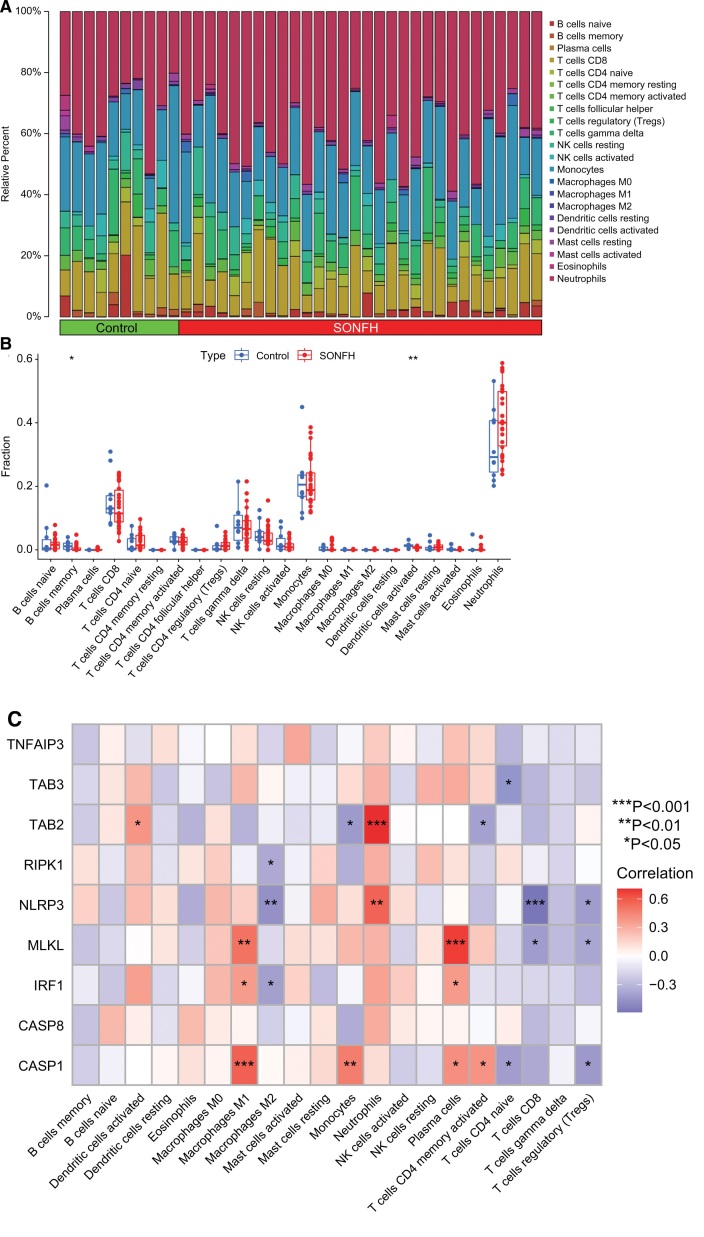
Correlation analysis between differentially expressed PRGs and immune infiltration. (A) Relative abundance of 22 immune cells between 2 groups. (B) Box plot of differential expression of immune infiltration between 2 groups. (C) Immune infiltration characteristics of differentially expressed PRGs (* *P* < .05, ** *P* < .01, *** *P* < .001).

Immunoinfiltration analysis was performed for all samples from the SONFH group and the control group. We analyzed the differences in the proportion of 22 immune cell types between the 2 groups based on the CIBERSORT algorithm (Fig. 2A). The results showed lower infiltration levels of memory B cells and activated dendritic cells in SONFH patients (Fig. 2B), which suggests that alterations in the immune system may contribute to the development of SONFH. In addition, we also analyzed differentiated PRGs associated with immune infiltration, and 7 genes (*TAB3, TAB2, RIPK1, NLRP3, MLKL, IRF1*, and *CASP1*) were remarkably associated with one or more of the 22 immune cells (Fig. 2C). This finding suggests that the infiltration state of immune cells may be a crucial link in the pathogenesis of PANoptosis involved in SONFH.

### 3.3. Identification of PANoptosis clusters in SONFH

**Figure 3.**
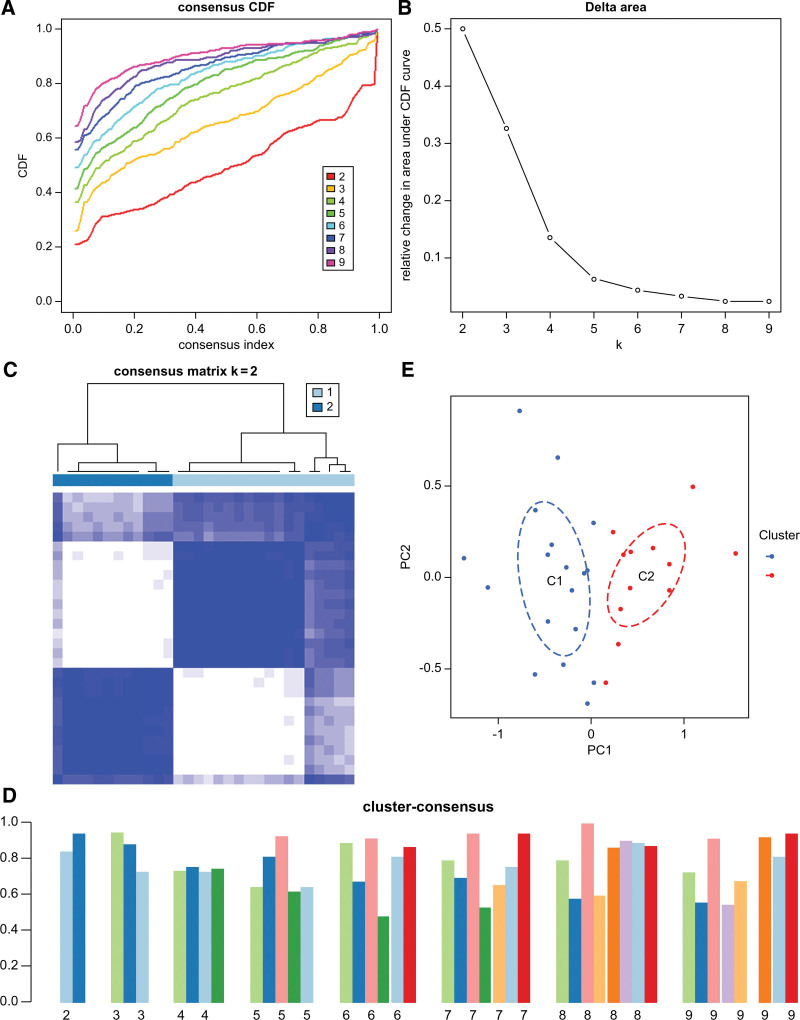
Identification of PANoptosis clusters in steroid-induced osteonecrosis of the femoral head (SONFH). (A) Cumulative distribution function showed a consistency distribution between K = 0.2–0.9; (B) Consensus clustering scores. (C) When K = 2, the discrimination of the consensus clustering matrix was more evident; (D) when K = 2, the consistency score of each cluster was the highest. © PCA scatter plot of cluster classification.

We classified the peripheral blood samples in the GSE123568 dataset according to the DEGs obtained. The consensus clustering matrix showed improved stability of the number of clusters, higher consistency scores for each subtype, and clearer discrimination when the k value was set to 2 (Fig. 3A–D). Principal component analysis scatter plots also revealed significant differences between the 2 clusters (Fig. 3E). Therefore, we divided the 30 SONFH patients into 2 clusters: C1 (n = 18) and C2 (n = 12).

### 3.4. Differentially expressed PRGs and differential analysis of immune cells between PANoptosis clusters

**Figure 4.**
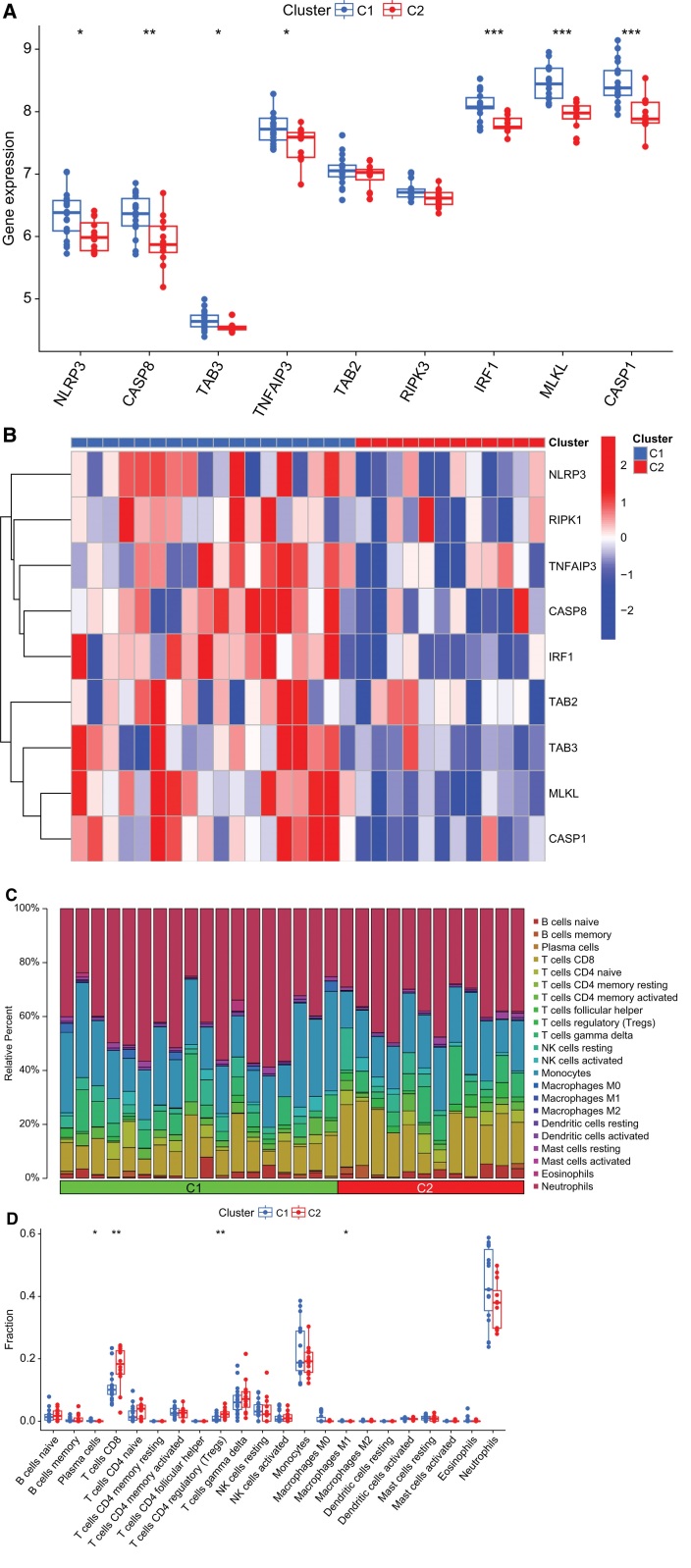
Differentially expressed PRGs and differential analysis of immune cells between PANoptosis clusters. (A) Boxplot of differentially expressed PRGs expression in 2 PANoptosis clusters. (B) Heatmap of differentially expressed PRGs expression in 2 PANoptosis clusters. (C) Relative abundances of 22 infiltrated immune cells in 2 PANoptosis clusters. (D) Boxplot of immune infiltration differences in 2 PANoptosis clusters. (* *P* < .05, ** *P* < .01, *** *P* < .001).

We evaluated the expression of differentially expressed PRGs in each cluster to further explore the molecular features between clusters. Figure 4A and B show the expression levels of differentially expressed PRGs in C1 and C2. C1 exhibited significantly higher expression levels of *IRF1, MLKL, CASP1, CASP8, NLRP3, TAB3*, and *TNFAIP3*, as shown in Figure 4A and B. Subsequently, immune infiltration analysis of samples was performed for each cluster. Results showed differences in the immune microenvironment between clusters. The proportion of plasma cells and M1 macrophages in C1 was relatively high. Meanwhile, the proportion of CD8 + T cells and Tregs was relatively high in C2 (Fig. 4C and D).

### 3.5. Gene module filtration and co-expression network construction

**Figure 5.**
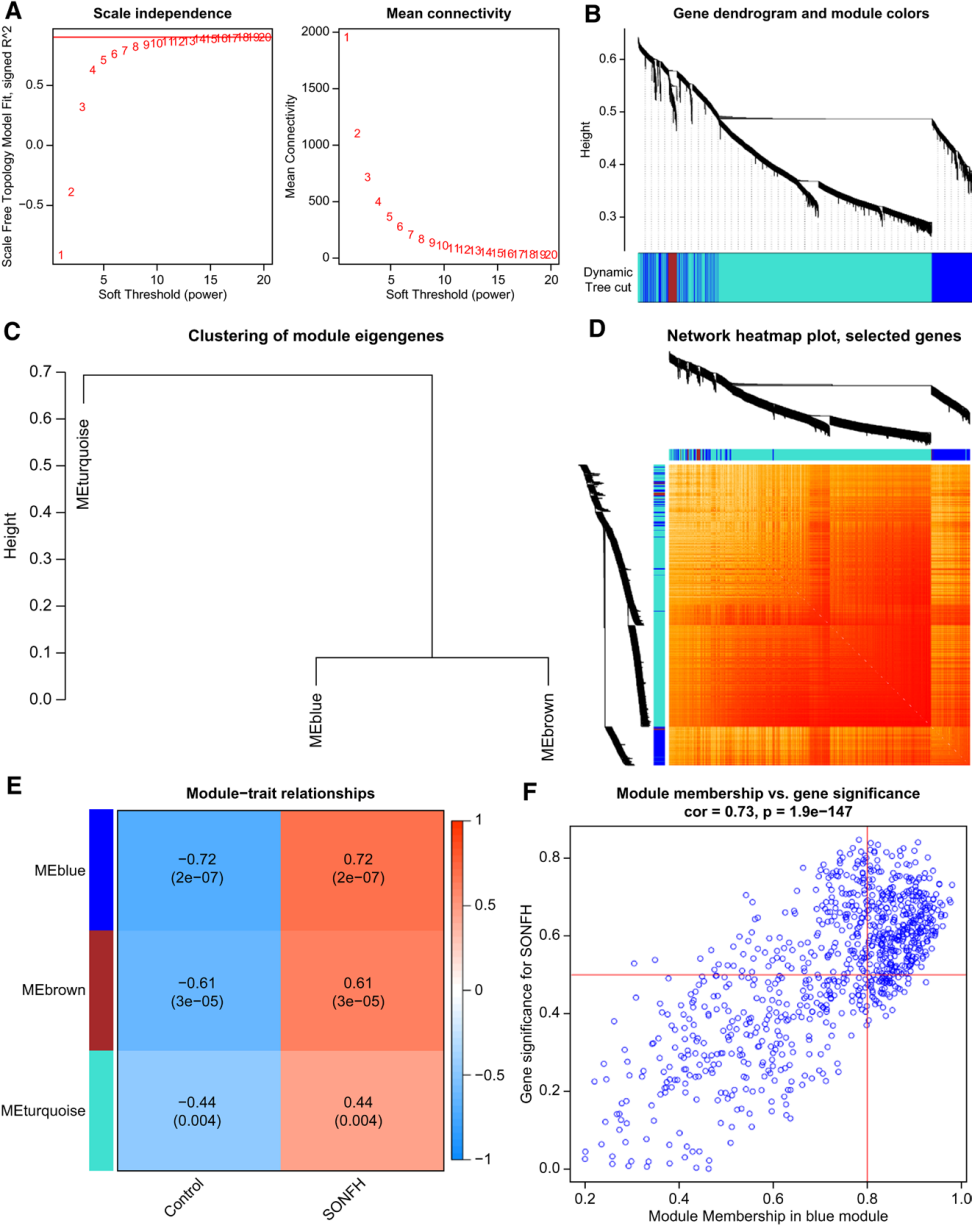
Co-expression network of differentially expressed PRGs in SONFH. (A) Determination of the optimal soft threshold. (B) Clustering dendrogram of genes. (C) Module eigengenes dendrogram. (D) Correlation heatmap between 3 modules. (E) Correlation analysis between module eigengenes and clinical status. (F) Scatter plot between the blue module membership and SONFH genes significance.

To identify key gene expression modules related to SONFH, we utilized the WGCNA algorithm to establish modules and co-expression networks between control subjects and SONFH patients. Next, we analyzed the top 25% of genes with the highest variation in the GSE123568 dataset. When the soft threshold was set to 12 and the scale-free fit index was equal to 0.9, a co-expressed network was built (Fig. 5A). We used the dynamic cutting algorithm to identify 3 different co-expression modules with different colors and generated a heatmap of the TOM (Fig. 5B–D). Then, we analyzed the relationship between the genes in the 3 modules and the clinical features of patients (control and SONFH). Finally, we found that the blue module, consisting of 439 genes, was most closely related to SONFH (Fig. 5E). In addition, we observed that the blue module memberships were positively correlated with gene significance (Fig. 5F).

**Figure 6.**
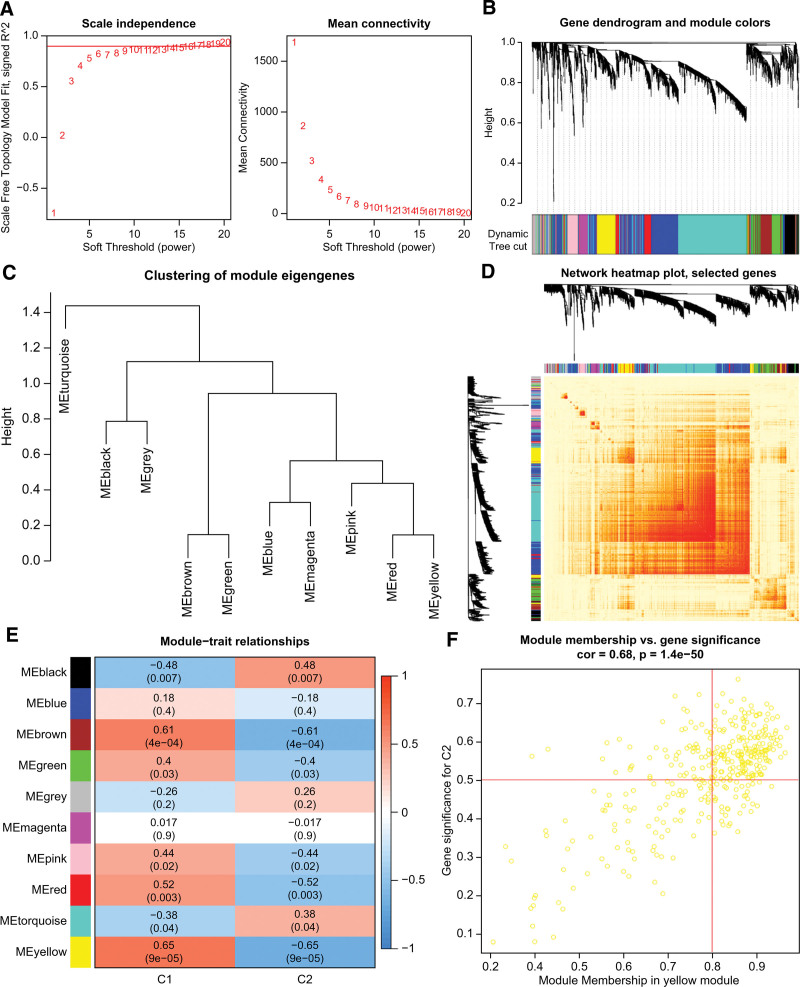
Co-expression network of differentially expressed PRGs in 2 PANoptosis clusters. (A) Determination of the optimal soft threshold. (B) Clustering dendrogram of genes. (C) Module eigengenes dendrogram. (D) Correlation heatmap between 10 modules. (E) Correlation analysis between module eigengenes and clinical status. (F) Scatter plot between the yellow module membership and C2 genes significance.

In addition, we used the WGCNA algorithm to analyze the key gene modules closely related to PANoptosis clusters. When we set the soft threshold value to 13 and the scale-free fit index to 0.9, we found it was most suitable to construct a co-expressed network (Fig. 6A). We divided the resulting 10 modules, containing 4709 genes, and presented a heatmap of the TOM (Fig. 6B–D). Analysis of module-clinical feature relationships (C1 and C2) revealed that the yellow module was highly associated with SONFH clusters (Fig. 6E). Correlation analysis showed a positive correlation between the yellow module membership and gene significance (Fig. 6F).

### 3.6. Screening of cluster-specific DEGs and biofunctional enrichment analysis

**Figure 7.**
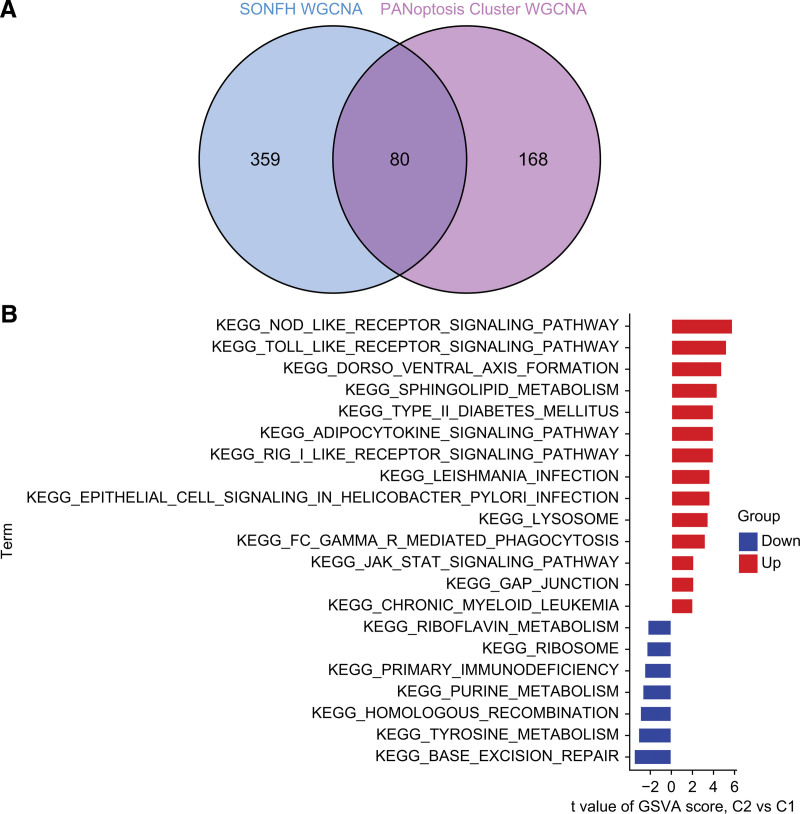
Screening of cluster-specific DEGs and biofunctional enrichment analysis between C1 and C2. (A) Intersection between the module genes of SONFH and the module genes of PANoptosis clusters. (B) Differences in characteristic signaling pathway activity between C1 and C2.

We identified 80 cluster-specific DEGs by analyzing the intersection of module genes in SONFH and C2 (Fig. 7A). Based on the cluster-specific DEGs, we further explored the different biological features between the 2 clusters using GSVA analysis. Our results showed that riboflavin metabolism, purine metabolism, and tyrosine metabolism were enhanced in C1, whereas Toll-like receptor signaling pathway, Nod-like receptor signaling pathway, adipocytokine signaling pathway, RIG-I-like receptors signaling pathway, and JAK/STAT signaling pathway were upregulated in C2 (Fig. 7B). Based on our findings, C2 may play a crucial role in regulating multiple immune responses.

### 3.7. Construction and evaluation of machine learning models

**Figure 8.**
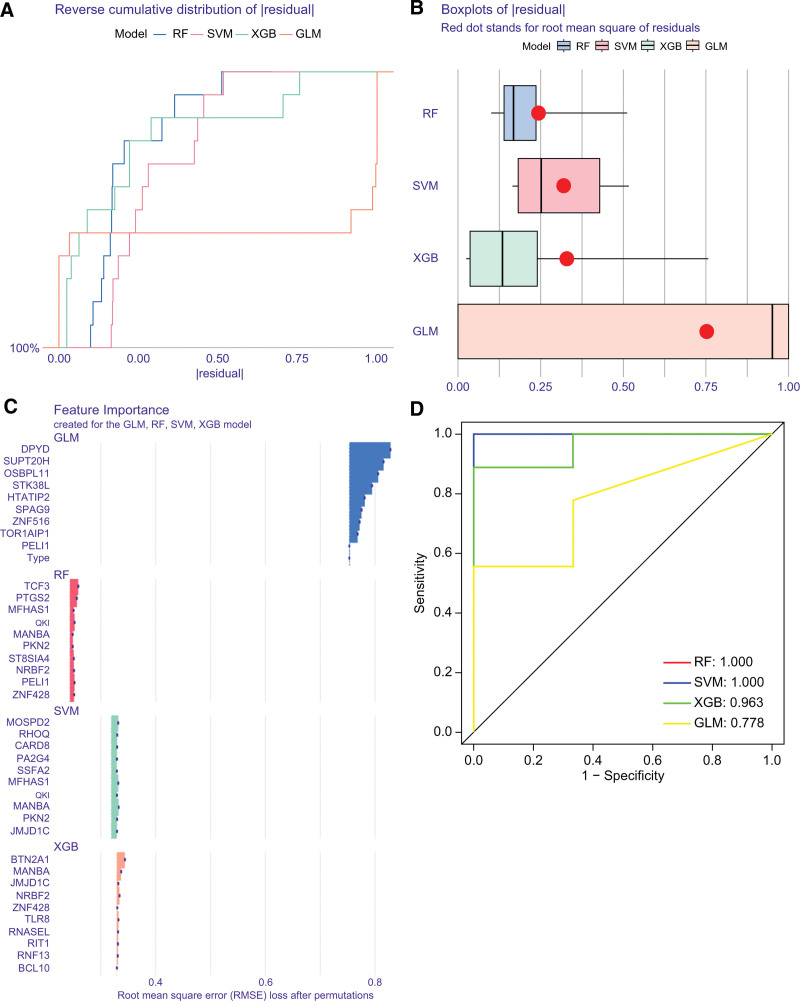
Construction and evaluation of generalized linear (GLM), support vector machine (SVM), eXtreme gradient boosting (XGB), and random forest (RF) models. (A) Distribution of cumulative residuals for the 4 models. (B) Boxplots of residuals of 4 models. (C) Feature importance genes in 4 models. (D) ROC curves of 4 models.

To select the key predictive genes with high diagnostic value, we built 4 machine learning models (GLM, SVM, XGB, and RF models) based on the 80 cluster-specific DEGs. We used the “Dalex” package to interpret the 4 models and plot the distribution of residuals in the test set for each model. The RF model demonstrated significantly lower residual errors compared to other models (Fig. 8A and B). We ranked the top 10 genes based on their feature importance scores calculated by the root mean square error for each model (Fig. 8C). Furthermore, ROC curves were plotted to evaluate the performance of each model, with RF and SVM models exhibiting the highest AUC (AUC = 1.000) (Fig. 8D). In summary, the RF model proved to be best able to distinguish between patients in different clusters. Finally, the most significant 5 genes (*ZNF428, PELI1, QKI, PTGS2*, and *TCF3*) were selected from the RF model as key prediction genes for further analysis.

**Figure 9.**
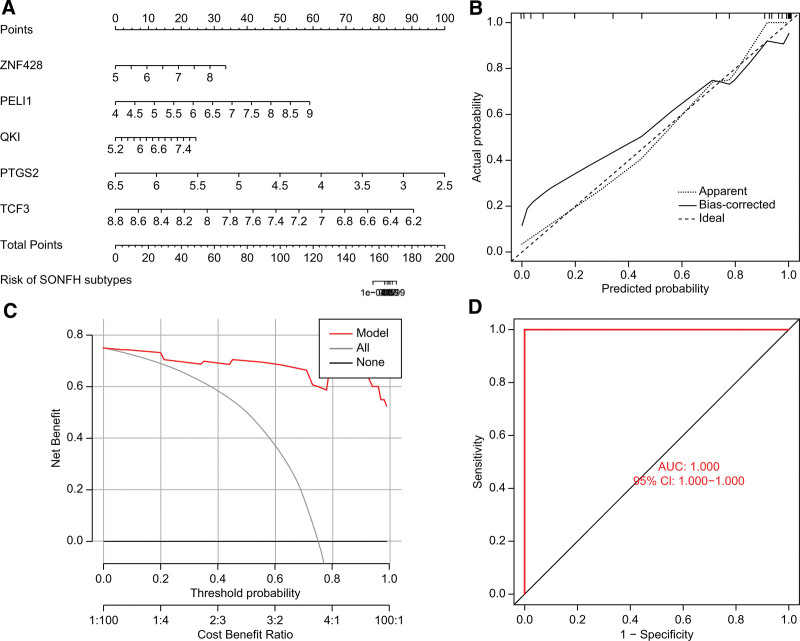
Construction and test results of the risk prediction model. (A) Construction of a nomogram for SONFH clusters. (B) Construction of a calibration curve. (C) Construction of DCA. (D) ROC curve test of the RF model based on the 5 key predictive genes.

We constructed a nomogram to estimate the risk of PANoptosis clusters in 30 SONFH patients to further evaluate the predictive efficiency of the RF model (Fig. 9A). Subsequently, we used decision curve analysis and calibration curves to evaluate the prediction efficiency of the nomogram model. Calibration curves showed that the error between predicted risk and actual SONFH cluster risk is small (Fig. 9B). Decision curve analysis showed that the nomogram model was accurate and helpful for clinical diagnosis (Fig. 9C). In addition, we tested our prediction model on a dataset of femoral head cartilage tissue, including SONFH patients and control subjects. The ROC curve showed that the prediction model performed satisfactorily. The AUC value in the GSE74089 dataset was 1.000 (Fig. 9D), indicating that the prediction model had substantial potential in distinguishing osteonecrosis of the femoral head patients from normal individuals.

### 3.8. Correlation analysis between differentially expressed PRGs and key predicted genes

**Figure 10.**
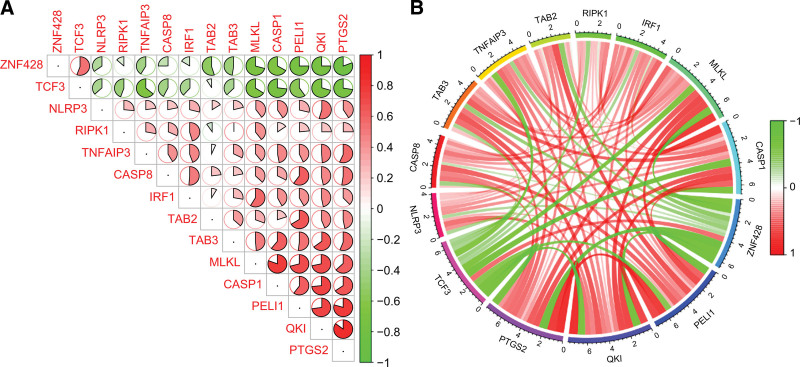
Correlation analysis between differentially expressed PRGs and key predicted genes. (A and B) Spearman correlation analysis between differentially expressed PRGs and key predicted genes.

We performed Spearman correlation analysis between differentially expressed PRGs and key predicted genes. The results showed that *ZNF428* had an antagonistic effect on *TAB2, MLKL*, and *CASP1*, whereas *TCF3* had an antagonistic effect on *TNFAIP3, CASP1*, and *MLKL. PELI1* had a strong synergistic effect on *CASP8, TAB2, TAB3, CASP1*, and *MLKL. QKI* had a strong synergistic effect on *NLRP3, TAB3, CASP1*, and *MLKL*. In addition, *PTGS2* had a strong synergistic effect on *TNFAIP3, CASP8, TAB3, CASP1*, and *MLKL*. Among them, *CASP1* and *MLKL* were significantly correlated with all 5 key predicted genes (Fig. 10A and B).

### 3.9. Drug target prediction of key predicted genes and their highly related PRGs

The aim of this study was to identify potential drugs for treating SONFH by mapping 5 key predictive genes and 2 highly correlated PRGs to the Coremine database. Using a significance criterion of *P* < .05, we selected the top 10 corresponding drugs based on the gene–drug interaction data available (Table 2). We have illustrated the most relevant graph for each gene in Figure S1, Supplemental Digital Content, http://links.lww.com/MD/M216.The screening of related gene–drug targets in this study will facilitate further clinical drug experiments on SONFH.

**Table 2:** Drug targets corresponding to key predictive genes and highly correlated PANoptosis-Related Genes.

Gene name	Screened drugs
*ZNF428*	Hepatitis B vaccine (*P* = .00110)
*TCF3*	Gambogic amide (*P* = 6.66E-4), SHP-1 agonist SC-43 (*P* = 6.66E-4), Sulindac sulfide (*P* = .00104), Sulfuretin (*P* = .00105), Cannabicyclol (*P* = .00110), CHOP regimen (*P* = .00132), Tubacin (*P* = .00152), Acetophenazine (*P* = .00157), Recombinant interleukin-6 (*P* = .00158), Diindolylmethane (*P* = .00176)
*PELI1*	Monocarboxylate transporter 1 inhibitor AZD3965 (*P* = 1.80E-4), Ethyl 6-(N-(2-chloro-4-fluorophenyl)sulfamoyl)cyclohex-1-ene-1-carboxylate (*P* = .00241), Methylene dimethane sulfonate (*P* = .00322), Lipopolysaccharide (*P* = .00399), Interleukin 1 receptor antagonist protein (*P* = .00441), Lysine (*P* = .00469), Recombinant interleukin-6 (*P* = .00697), Recombinant tumor necrosis factor family protein (*P* = .00833), Pegademase bovine (*P* = .00975), Denenicokin (*P* = .0115)
*QKT*	Pan-RAF inhibitor LY3009120 (*P* = 1.32E-4), JAK2 inhibitor AZD1480 (*P* = 4.10E-4), Purmorphamine (*P* = 8.15E-4), Nattokinase (*P* = .00104), Entrectinib (*P* = .00127), Olomoucine (*P* = .00148), Trametinib (*P* = .00173), Dexrazoxane (*P* = .00391), Leptomycin B (*P* = .00496), Hyperoside (*P* = .00538)
*PTGS2*	N-(2-cyclohexyloxy-4-nitrophenyl)methanesulfonamide (*P* = 1.61E-6), Dinoprostone (*P* = 2.07E-6), Celecoxib (*P* = 3.36E-6), Rofecoxib (*P* = 4.82E-6), Nonsteroidal anti-inflammatory drug (*P* = 8.97E-2), Arachidonic acid (*P* = 1.60E-5), Nimesulide (*P* = 2.00E-5), 1-((4-Methylsulfonyl)phenyl)-3-trifluoromethyl-5-(4-fluorophenyl)pyrazole (*P* = 2.04E-5), Valdecoxib (*P* = 3.92E-5), Recombinant interleukin-1-beta (*P* = 4.04E-5)
*MLKL*	3-Methyladenine (*P* = 7.71E-4), Cytarabine (*P* = .00421), Cytosine (*P* = .00541), Dextrans (*P* = .00881), Aspartic acid (*P* = .00941), Sirolimus (*P* = .00974), Adenine (*P* = .0116), Myeloperoxidase (*P* = .0140), Cysteine (*P* = .0205), Recombinant interleukin-6 (*P* = .0369)
*CASP1*	Iboctadekin (*P* = 6.11E-7), Interleukin-1 beta-converting enzyme inhibitor (*P* = 9.39E-7), Recombinant interleukin-1-beta (*P* = 1.88E-6), Belnacasan (*P* = 4.95E-6), L 709049 (*P* = 8.72E-6), Nigericin (*P* = 2.37E-5), Lipopolysaccharide (*P* = 4.48E-5), Recombinant interleukin-6 (*P* = 6.03E-5), Pralnacasan (*P* = 6.88E-5), Interleukin 1 receptor antagonist protein (*P* = 1.01E-4)

### 3.10. Validation of key PRGs

**Figure 11.**
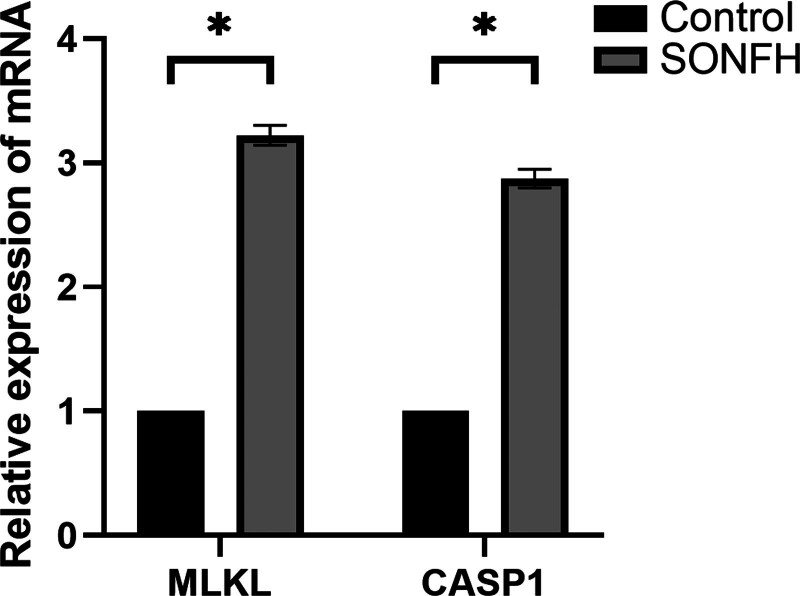
Validation of 2 PRGs using RT-qPCR. Relative mRNA expression of *CASP1* and *MLKL* in human (*P < .05).

To confirm our results, we verified 2 PRGs highly correlated with key predicted genes by RT-qPCR. We observed a significant upregulation of CASP1 and MLKL expression in human peripheral blood samples from SONFH patients compared to controls (Fig. 11). The results are consistent with our predictions derived using bioinformatics tools.

## 4. Discussion

With advancements in medicine, hormones have become increasingly prevalent in clinical practice. A major contributing factor to SONFH is long-term or high-dose glucocorticoid use. Studies have shown that 9% to 40% of people with glucocorticoid use develop SONFH.^[17]^ The occurrence and development of SONFH is a dynamic pathological process regulated by multiple factors, links, stages, and genes. Although the pathological mechanism of SONFH is related to the imbalance of the regulation mechanism of the homeostasis of the bone microenvironment in the femoral head, its specific mechanism remains unclear. Under the action of glucocorticoids, the activation of osteoregulatory transcription activators is blocked. In addition, the differentiation of pre-osteoblasts into mature osteoblasts is inhibited, the mineralization process of the bone matrix is hindered, and the proliferation activity and osteogenic ability of osteoblasts are weakened.^[18]^ The above mechanisms result in an imbalance of bone microenvironment homeostasis, which eventually leads to abnormal bone remodeling in the femoral head, local sclerosis, and cystic degeneration, resulting in or aggravating the collapse of the femoral head.^[19]^ Therefore, understanding the pathological mechanism of glucocorticoids in SONFH and exploring specific biomarkers for early diagnosis are of significant theoretical and clinical significance. Furthermore, although cell death usually occurs in mixed forms under pathological conditions due to extensive crosstalk, its relationship with SONFH remains unclear.^[20]^ Current experimental evidence suggests that the regulation of PANoptosis, caused by certain infections and cellular stress, is complex and diverse. However, this complex regulation is achieved by PANoptosome. PANoptosome, a multiprotein complex that provides a molecular scaffold, contains key proteins that activate pyroptosis, apoptosis, and necroptosis.^[8]^ These apoptosome complexes promote the activation of downstream cell death effector molecules, release of key proteins that activate pyroptosis (NLRP3), apoptosis (CASP8), and necroptosis (RIPK1/RIPK3 complex), leading to lytic inflammatory cell death.^[6]^ Currently, the mechanism of PANoptosis in SONFH has not been further studied. Therefore, the aim of this study was to elucidate the mechanism of PRGs in the SONFH phenotype and immune microenvironment, and predict SONFH subtypes based on their expression levels.

In this study, we have provided the first comprehensive analysis of the expression profile of PANoptosis regulators in the peripheral blood of SONFH patients and control subjects. Compared with the normal population, we observed significant dysregulation of several PRGs in SONFH patients, suggesting their potential involvement in the pathogenesis of SONFH. Subsequently, we performed correlation analysis among differentially expressed PRGs and found a significant synergistic effect between some PANoptosis regulators. Immune infiltration was also altered between SONFH patients and control subjects. SONFH patients showed lower levels of memory B cells and activated dendritic cell infiltration. To further explore the heterogeneity of PRG dysregulation in SONFH patients, we performed cluster typing and identified 2 distinct subtypes (C1 and C2), characterized by differential expression of specific PRGs and distinct immune infiltration patterns. According to the correlation analysis results of 9 differentially expressed PRGs and immune infiltration, *IRF1, MLKL, CASP1, CASP8, NLRP3, TAB3*, and *TNFAIP3* were significantly highly expressed in C1, and the proportion of plasma cells and M1 macrophages was relatively high. In contrast, within C2, the aforementioned differentially expressed PRGs exhibited markedly reduced expression, whereas the proportion of CD8 + T cells and Tregs was comparatively elevated. This suggests that the immune-modulating effects of these PANoptosis-related genes vary in different subtypes of SONFH. Our GSVA analysis showed that C1 was mainly enriched in the biological processes of riboflavin, purine, and tyrosine metabolism. C2 was mainly enriched in pathways related to immune cell activation and differentiation (Toll-like receptor, Nod-like receptor, and RIG-I-like receptor signaling pathways). Studies have demonstrated that signaling pathways play an important role in the activation and differentiation of T cells.^[21]^ In summary, our findings suggest that C2 may have a higher level of T cell activation, which would contribute to the pathological processes of SONFH.

In this study, we compared the prediction efficacy of 4 machine learning models (GLM, SVM, XGB, and RF) constructed based on 80 cluster-specific DEGs and finally selected the RF model. This model showed the highest predictive efficacy (AUC = 1.000), which indicated that the RF model had satisfactory efficacy in predicting SONFH subtypes. We then selected 5 key predictive genes (*PELI1, QKI, PTGS2, TCF3*, and *ZNF428*) to construct the RF model based on them. Pellino 1 (PELI1), a member of the Pellino family, is an E3 Ubiquitin Ligase involved in mediating TLR3/TLR4 signaling.^[22,23]^ Studies have shown that PELI1 is a key factor in tumor necrosis factor alpha (TNF-α)-mediated cell death pathways, which inhibit apoptosis and promote necroptosis.^[24]^ In addition, PELI1 can also promote apoptosis-associated speck-like protein containing a CARD (ASC) spot formation and oligomerization, by mediating the ubiquitination of the K63 linker of ASC, thereby promoting inflammasome activation. PELI1 was identified as a crucial regulator of NLRP3 inflammasome activation and involved in the inflammatory response.^[25]^ Therefore, we suggest that PELI1 may be involved in regulating pyroptosis, apoptosis, and necroptosis caused by upregulated inflammasomes in osteoblasts in SONFH. Quaking (QKI) is an RNA-binding protein with a variety of biological functions, such as regulating mRNA expression, inhibiting tumors, and ameliorating inflammation.^[26]^ Knocking out *QKI* from myeloid cells leads to the infiltration of pro-inflammatory cytokines into the bone marrow cavity and inhibition of osteoblast formation, suggesting that QKI deficiency may contribute to abnormal bone remodeling in SONFH.^[27]^ In our study, we adopted the Weighted Gene Co-expression Network Analysis (WGCNA) method to select genes associated with PANoptosis regulators, which is one of the innovative aspects of our research. Unlike the commonly seen m6A-related factors in previous studies, we focused on exploring PANoptosis regulators. Through WGCNA, we revealed the associations between these factors and steroid-induced osteonecrosis of the femoral head (SONFH). This method enabled us not only to construct a co-expression network among genes but also to discover novel genes related to SONFH. Therefore, we suggest that QKI may be involved in the abnormal immune microenvironment in SONFH, which in turn affects the activity of osteoblasts in the femoral head, leading to or worsening the collapse of the femoral head. Prostaglandin endoperoxide synthase 2 (*PTGS2*), also known as *COX*-2, encodes a key enzyme in the biosynthesis of prostaglandins. It has dual roles as a dioxygenase and a peroxidase. Jiang et al^[28]^ showed that insulin-like growth factor 1 (IGF-1) upregulates the expression of COX-2 through the PI3K/AKT pathway, which reverses the inhibitory effect of dexamethasone on bone morphogenetic protein 9 (BMP9)-induced osteogenic differentiation of mouse embryonic fibroblasts. Therefore, *PTGS2* may be a potential therapeutic target for SONFH patients due to its role in prostaglandin biosynthesis and inflammation. TCF3 belongs to the basic helix-loop-helix (bHLH) protein family.^[29]^ TCF3 proteins form heterodimers or homodimers with other bHLH proteins to perform tissue- or cell-type-specific functions.^[30]^ Liu et al^[31]^ found that TCF3 has been proved to be a positive regulator of osteogenic differentiation. As a key transcription factor of the classical Wnt pathway, it promotes osteogenic differentiation in vitro and ectopic bone formation in vivo. In addition, their results showed that miR-17 could modulate the opposite effects of the classical Wnt signaling pathway on osteogenic differentiation by directly targeting TCF3 in different microenvironments. Therefore, TCF3 may be a promising target for further research on the treatment of SONFH. Zinc finger protein 428 (*ZNF428*) is a protein-coding gene whose biological function in orthopedic-related diseases is rarely reported. In this study, we screened *ZNF428* and found its expression to be dysregulated in SONFH. Further exploration of its biological significance in SONFH could provide insights into the pathogenesis of SONFH and potential therapeutic targets.

Subsequently, the RF model based on the 5 key predictive genes was validated in the external dataset GSE74089 (AUC = 1.0), confirming its effective diagnostic performance. More importantly, a nomogram model was constructed for the diagnosis of SONFH subtypes using *PELI1, QKI, PTGS2, TCF3*, and *ZNF428*. We found that this model has significant predictive power and clinical application value. Evidently, there is merit in using it for evaluating the pathological diagnosis of SONFH patients and SONFH subtypes.

To further explore the relationship between the differentially expressed PRGs and the key predicted genes derived from the RF model, correlation analysis was performed between them. The results showed that *CASP1* and *MLKL* were significantly correlated with each of the 5 key predicted genes. *CASP1* and *MLKL* had a strong antagonistic effect on *TCF3* and *ZNF428* but a strong synergistic effect on *PELI1, QKI*, and *PTGS2*. CASP1 (cysteine aspartic protease 1), as a component of the PANoptosome, is known to play a major role in PANoptosis, and inhibitors of caspase-1 can inhibit both apoptosis and pyroptosis pathways in PANoptosis to reduce cell death.^[32]^ Studies have shown that excessive use of glucocorticoids can not only induce osteoblast apoptosis^[33]^ but also render the femoral head tissue more susceptible to oxidative stress and reactive oxygen species (ROS) production.^[34]^ However, excessive ROS levels will over-activate NLRP3 and upregulate the expression of the caspase-1/GSDMD pathway, leading to pyroptosis and dysfunction of osteoblasts.^[12]^ In addition, the PANoptosome releases the RIPK1/RIPK3 complex upstream, which promotes the phosphorylation of mixed lineage kinase domain like pseudokinase (MLKL) and the formation of MLKL protein, leading to cell necrosis.^[35]^ Fan et al^[36]^ established a TNF-α-induced ONFH model in human bone microvascular endothelial cells in vitro and found that necroptosis was activated through the RIP1/RIP3/MLKL signaling pathway, leading to increased necroptosis of human bone microvascular endothelial cells and aggravation of osteonecrosis. In summary, we suggested that *CASP1* and *MLKL* may influence the involvement of PANoptosis in SONFH through mutual regulation between them and the 5 key predicted genes.

Finally, the 5 key predictive genes and 2 highly related PRGs were mapped to the Coremine medical database, and corresponding drug targets were obtained, providing direction for further research on the treatment of SONFH.

Nevertheless, our study has limitations. First, it relies on bioinformatics analysis, and further experimental or clinical studies are required to validate our predicted targets. Second, additional SONFH datasets are needed to verify the accuracy of PANoptosis clustering. Furthermore, the potential relationship between PRGs and immune response requires further exploration. Lastly, although we verified the differential expression of key PRGs between SONFH patients and controls by RT-qPCR, further experiments are necessary to elucidate the underlying mechanisms.

## 5. Conclusion

We identified variations in immune cell profiles between SONFH and control subjects and elucidated differences in immune infiltration among SONFH patients with different PANoptosis clusters. The RF model, based on 5 key predictive genes as the optimal model, accurately evaluates the risk of SONFH patients and different subtypes of SONFH. In summary, our study explored the influence of PANoptosis on SONFH and investigated potential molecular mechanisms underlying differences in SONFH. The relevant drug targets were also identified, which provide a direction for further research on the treatment of SONFH.

## Acknowledgments

We would like to thank Editage (www.editage.cn) for English language editing.

## Author contributions

**Conceptualization:** Qiang Ding.

**Data curation:** Jinfu Liu, Xiangbin Rong, Zhao Tian, Limin Chen, Hongcheng Tao, Hao Li.

**Formal analysis:** Jinfu Liu, Xiangbin Rong, Zhao Tian, Limin Chen, Hongcheng Tao, Hao Li.

**Funding acquisition:** Ping Zeng.

**Methodology:** Qiang Ding.

**Visualization:** Qiang Ding, Bo Xiong.

**Writing – original draft:** Qiang Ding, Bo Xiong.

**Writing – review & editing:** Qiang Ding, Bo Xiong.

## Supplementary Material


