# Supplementary figures and images for: Bioinformatics analysis of PANoptosis regulators in the diagnosis and subtyping of steroid-induced osteonecrosis of the femoral head
Source: Medicine (Baltimore). 2024 May 3;103(18):e37837. doi: 10.1097/MD.0000000000037837 (PMC11062652; doi:10.1097/MD.0000000000037837)

Most Significant Screened Drug for Each Gene

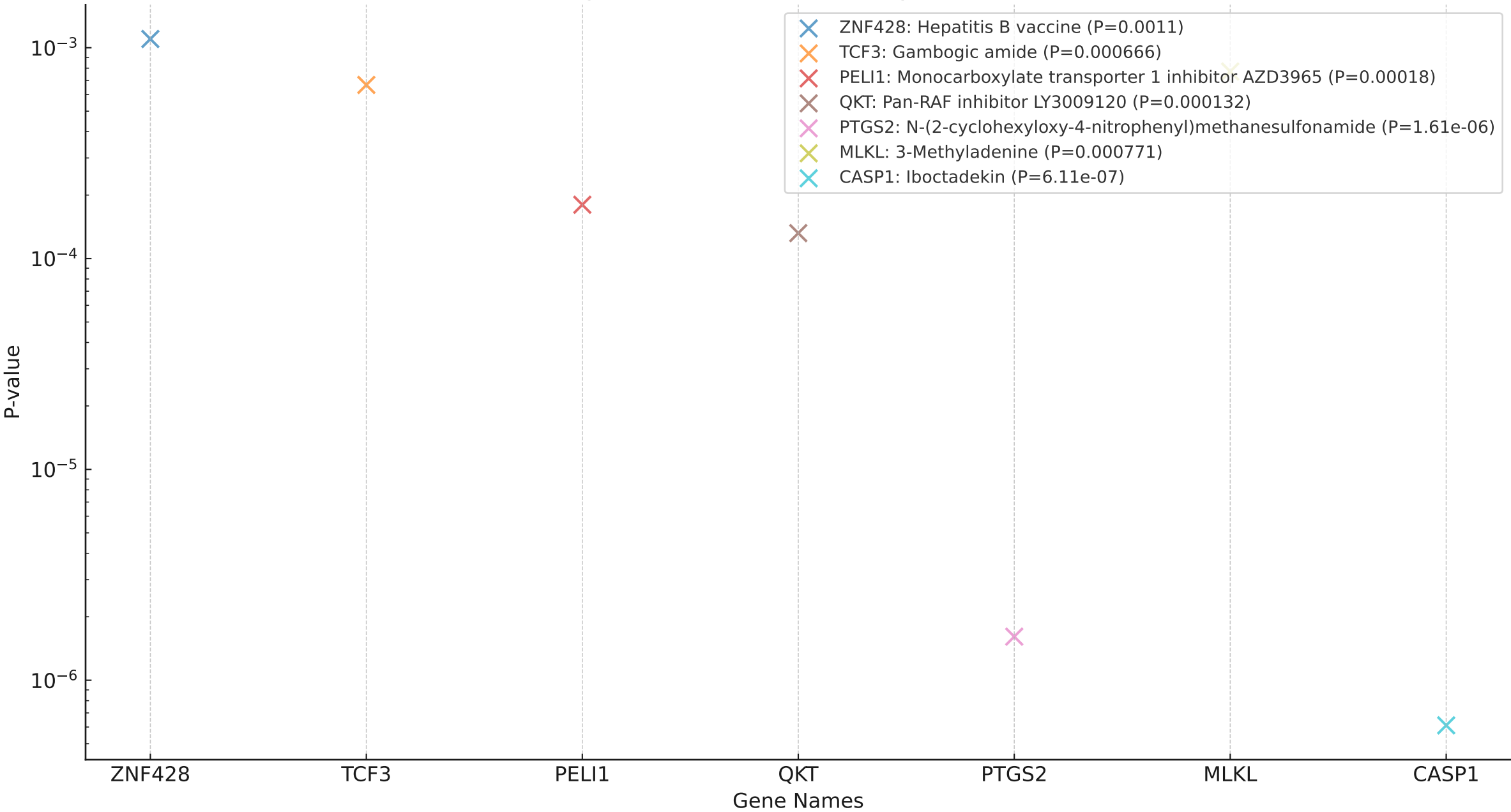

Supplement: Supplementary file 1 [file medi-103-e37837-s001.pdf]
